# Information and Communication Technologies (ICTs) enabling integrated primary care for complex patients: a protocol for a scoping review

**DOI:** 10.1186/s13643-022-02057-5

**Published:** 2022-09-07

**Authors:** Farah Tahsin, Alana Armas, Apery Kirakalaprathapan, Heather Cunningham, Mudathira Kadu, Jasvinei Sritharan, Carolyn Steele Gray

**Affiliations:** 1 Institute of Health Policy, Management, and Evaluation, University of Toronto, Toronto, Canada; 2 Bridgepoint Collaboratory for Research and Innovation, Lunenfeld-Tanenbaum Research Institute, Sinai Health, Toronto, Canada; 3 School of Public Health and Health Systems, University of Waterloo, Kitchener, Canada; 4 Gerstein Science Information Centre, University of Toronto, Toronto, Canada

**Keywords:** Patients with complex chronic conditions, Integrated primary care, Information and communication technologies, Primary care, Multi-morbidity

## Abstract

**Introduction:**

An increasing number of individuals are living with multiple chronic conditions, often combined with psychosocial complexities. For these patients with complex conditions, an integrated primary care model that provides care coordination and a team-based approach can help manage their multiple needs. Information and communication technologies (ICTs) are recognized as a critical enabler of integrated primary care. A better understanding of the use of ICTs in an integrated care setting and how ICTs are being leveraged would be beneficial to identify knowledge gaps and could lead to successful implementation for ICT-based interventions.

**Objective:**

This study will systematically scope the literature on the topic of ICT-enabled integrated healthcare delivery models for patients with complex care needs to identify which technologies have been used in integrated primary care settings.

**Method:**

This study protocol outlines a scoping review of the peer-reviewed literature, using Arksey and O’Malley’s (enhanced by Levac et al.) scoping review methodology. Peer-reviewed literature will be identified using a multi-database search strategy. The results of the search will be screened, abstracted, and charted in duplicate by six research team members.

**Discussion:**

The key findings of the study will be thematically analyzed to describe the implemented ICTs aimed for complex patients within the integrated primary care model. The finding will highlight what types of ICTs are being put in place to support these models, and how these ICTs are enabling care integration. This review will be the first step to formally identify how ICT is used to support integrated primary health care models. The results will be disseminated through peer-reviewed publications, conference presentations, and special interest groups.

**Supplementary Information:**

The online version contains supplementary material available at 10.1186/s13643-022-02057-5.

## Background

Across Canada and globally, there is a growing number of individuals living with one or more chronic conditions, alongside of additional psychosocial challenges and socioeconomic factors [1].. As of 2021, 33% of Canadians live with two or more chronic conditions [2]. This patient population often referred to as patients with complex conditions, tends to experience disability, functional decline, poor quality of life, and are among the highest users of the healthcare system [3]. This high healthcare usage can cause economic burden on health system [4]. This patient group also reports higher levels of dissatisfaction with their care compared with less complex counterparts [5]. As health systems globally are experiencing a growth in the population of patients with complex health needs [6], many are seeking to shift from old disease-centric models, focusing on treating illnesses in isolation, towards person-centered and integrated models focusing on treating the whole person [7, 8]. Recent study findings suggest that being able to access person-centered health and social care from providers with whom they have a positive and respectful relationship can improve multi-morbid patients’ care experience [9].

Primary health care is a crucial starting point in this movement towards whole-person care as it is often patients’ first contact of care in their community and where they receive continuous, comprehensive care [10]. In the last decade, Wagner’s Chronic Care Model (CCM) [11] has been widely used to design coordinated primary health care to resolve the issue of fragmented care when managing chronic conditions. CCM was developed through a review of interventions tailored for patients with chronic conditions [12]. The validity of CCM has been confirmed through Cochrane Collaboration reviews [13] as well as in multiple primary care settings among patients with chronic conditions [14–16]. CCM aims to promote care for individuals with chronic conditions that encompass community and health care services. With the recent expansion in the adoption of eHealth technologies to support care delivery, an eHealth enhanced CCM model (eCCM) has been proposed. eCCM has also been developed through literature review [17]. Although eCCM has not been validated through empirical evidence yet, it provides an organizing framework to explore the typologies of ICTs used to support patients with chronic conditions in the primary care setting, which makes this model highly relevant for this scoping review.

eCCM model acknowledges the importance of information and communication technologies (ICTs) in enabling communication between patients and care providers as part of chronic care management [17–19]. ICT is a broad term. For this study, we follow the World Health Organization (WHO)’s the definition of ICT [20], which is health services and information delivered by the combined use of the internet and other electronic communication technologies [21, 22].. ICT encompasses multiple health technologies such as telehealth, telemedicine, mobile health-based interventions, etc. By using ICTs patients are empowered with information and their care team is better engaged and proactive, which leads to more productive interactions between patients and their primary care teams [17]. The CCM and eCCM are useful for helping understand integration in support of chronic disease management for individual patients. However, these individual-level interactions are often couched within a larger system context.

Valentijn et al.’s Rainbow model of Integrated Care (RMIC) [23] helps to understand the wider context of integrated care, taking a population-health perspective to identify how health and social care can be integrated at clinical, professional, organizational, and system levels. RMIC was initially developed through a literature review as well as expert consultation [23]. RMIC has been validated in multiple integrated primary care settings and among chronic patient populations [23–25]. While the emphasis of the CCM is on the provider and patient, the RMIC acknowledges other key players in the delivery of integrated care such as system and policy-level stakeholders in roles that include governance, finance, and other decision-makers linked to health systems. RMIC considers ICTs as valuable in improving the comprehensiveness of care and communication within and outside of health systems to deliver person-centered care and improve care continuity for patients with complex conditions. Table 1 describes the key elements of eCCM and RMIC.

**Table 1:** Key elements of the Rainbow integrated Model of Care (RMIC) and the eHealth enhanced chronic care model (eCCM)

Concept	Key elements	Example
RMIC [23]	**Clinical integration:** Refers to the coordination of person-focused care in a single process across time, place and discipline	The care model supports seamless individual care planning, individual case management, etc.
	**Professional integration:** Inter-professional partnerships and coordination of services across multiple disciplines	The care model supports multi-disciplinary care within and between the organizations (i.e., general physician and community nurse)
	**Organizational integration:** Inter-organizational coordination of services across multiple organization (e.g., contracting strategic alliances, knowledge networks, mergers), including common governance mechanisms to deliver comprehensive services to a defined population	The care model is supported by organizational integration such as coordination of services between long term care and primary health care
	**System integration:** A horizontal and vertical alignment of rules and policies within a system	The care model is within a structured integrated care model network such as the Veterans Health Administration [26]
	**Functional integration:** Refers to how the key support functions (i.e., financial, back office, and Information management system) are coordinated	The care model is situated in an integrated network where financial, information, and management system are coordinated (i.e., federal funding allotted in the network)
	**Normative integration:** Refers to the maintenance of coherent and shared mission, vision, and culture between disciplines, external stakeholders, and organizations.	The care model is placed in an integrated network where a mutual aim/vision is shared across stakeholders (i.e., reduce wait time)
eCCM [17]	**eCommunity resources**: Refers to developing strategies that link with community organizations and virtual health-related eCommunities	Health-related social networks and virtual communities that facilitate care connections
	**Health systems enhancements:** Refers to the strategies in place to support patient engagements and self-management support	Web-based health platforms, mobile health that support quality improvement
	**Delivery system design enhancements:** Refers to the systems in place to promote teamwork practice to deliver care efficiently	Electronic Health Records (EHRs), web-based health platforms that facilitate information sharing
	**Self-management support enhancements:** Refers to the patients’ active role in managing their care	Health apps and online resources that support patients’ self-management skills
	**Clinical decision support enhancements:** Refers to providers and health consumers’ access to evidence-based clinical guidelines, protocols, care standards, and self-management resources to make an informed decision	Online platforms, EHRs to virtually access protocols and guidelines
	**Clinical information systems enhancements:** Refers to managing information systems (i.e., patient databases, patient portals/personal health records) to facilitate efficient care	mHealth apps and online platform to coordinate care and monitor patients’ health status

Previous studies have identified that ICTs can enable care integration in the primary care models through various mechanisms such as information sharing across interdisciplinary team members and enabling synchronous/asynchronous patient-provider communication [27, 28]. However, there are currently no reviews available to document what ICTs are being put in place to support these models, and how these ICTs are enabling the process of care delivery. This scoping review seeks to bring together the current research in this space to provide a more extensive overview of the state of ICT in support of integrated primary health care delivery for patients with complex care needs. By scoping the literature we hope to identify promising approaches and current gaps that could help to guide future research, technology development, and implementation in this space.

### Study objective

The objective of this scoping review is to systematically scope the literature on the topic of ICT-enabled integrated primary care delivery models for patients with complex conditions in order to (1) map how technology is used to support patients with complex conditions in integrated primary care models and (2) identify specific characteristics and features of ICTs that enable key activities of integrated care delivery.

## Methods

For this scoping review, we will follow Arksey and O’Malley’s [29] scoping review framework that was enhanced by Levac et al. [30] (Table 2). Each of these stages is described in detail below.

**Table 2:** Scoping review stages

1. Identifying the research question	
2. Identifying relevant literature	
3. Study selection	
4. Charting the data	
5. Collating, summarizing and reporting findings	
6. Consulting and translating knowledge	

### Stage 1: Identifying the research question

The primary research question for this study is:

“What are the information communication technologies that are used in the delivery of integrated primary care for patients with complex care needs?”

To operationalize the guiding research question, we have added the following sub-questions:

1. Which information communication technologies (e.g., electronic medical records, virtual care, telehealth, monitoring, sensors, patient portals) are being used for patients with complex care needs? What are the functionalities and characteristics of these technologies?
2. How are these technologies being used in the integrated primary care model in terms of care integration?
3. What factors are associated with a successful implementation of ICTs in the integrated primary care models?

To answer the research questions, the definitions outlined in Table 3 below were used to guide the development of the major concepts employed in the search strategy.

**Table 3:** Definitions and operationalized search strategies of eHealth, integrated care, patients with complex chronic needs primary health care

Concept	Definition	Search strategy for each domain
eHealth [21, 22]	Health services and information delivered by the combined use of the internet and other electronic communication technologies. This is a broad definition of eHealth. Thus, eCCM model is used to categorize eHealth based on the primary functions of a technology (i.e., decision support).	exp Technology/ OR exp Medical Records Systems, Computerized/ OR exp informatics/ OR exp computing methodologies/ OR exp Technology, Radiologic/ OR exp Telecommunications/ OR exp Management Information Systems/ OR exp Diagnosis, Computer-Assisted/ OR ((communicat* or health* or informat* or comput* or medical) adj3 (technol* or system* or applicat* or process*)).tw,kf. OR(electronic adj3 record*).tw,kf. OR(ehealth or electronic health or telehealth or tele-health or telemedicine or tele-medicine or telecommunicat* or tele-communicat* or videoconferenc* or video-conferenc* or virtual care or teleradio* or tele-radio* or telemetry or mobile app* or informatics or computer-assist* or computer assist* or mobile health or mhealth or m-health or software or EHR? or EMR?).tw,kf. ((virtual or remote or distance or mobile or video) adj3 (consult* or health or medicine)).tw,kf.
Integrated care model [23]	A coordinated collaborative, multi-disciplinary (two or more health professionals involved) and person-centered care delivery system. In this review, an integrated care model is defined according to Valentijn’s Rainbow Model of Integrated Care because of the model’s broader focus on integrated care.	exp "Delivery of Health Care, Integrated"/ OR "Continuity of Patient Care"/ OR exp Patient Care Planning/ OR exp Patient-Centered Care/ OR exp Patient Care Management/ OR Patient Care/ ORexp Interprofessional Relations/ OR ((Integrat* or multidisciplin* or interdisciplin* or interprofession* or team* or coordinat* or comprehensive or shar* or manage* or organi?ed or coop* or seamless or continu*) adj3 (care or healthcare or service* or deliver* or communicat* or relation* or treatment* or strateg* or program* or system*)).tw,kf. OR Team*.tw,kf. OR ((Case or cases or care or transition* or patient* or disease* or treatment*) adj3 (manage* or plan*)).tw,kf. OR(patient adj3 (cent?ed or tailored or integrat* or orient* or focus*) adj3 care).tw,kf. OR ((Linked or network* or structur*) adj3 care).tw,kf. OR (Care adj3 (coordinat* or continu* or guid* or transmural)).tw,kf. OR ((Critical or clinical) adj2 pathway*).tw,kf.
Patients with complex care needs [1]	Individuals with multiple chronic conditions, often aggravated by additional psychosocial challenges. The complexity of their conditions impacts treatment, health outcomes, and quality of life.	exp Chronic Disease/ OR exp Comorbidity/ OR ((chronic* or complex or multi* or concurren* or co-occur* or co occur* or co-exist* or co exist* or dual or permanent or nonrevers* or non-revers*) adj2 (diagnos* or disease* or ill* or condition* or insufficienc* or disorder* or sick*)).tw,kf.OR (multimorbid* or multi-morbid* or comorbid* or co-morbid* or CCC).tw,kf. OR (poly-patholog* or polypatholog*).tw,kf. OR (pluri-patholog* or pluripatholog*).tw,kf.
Primary health care [10]	The first point of contact that includes key elements such as “disease prevention, health promotion, population health, and community development within a holistic framework, to provide essential community-focused health care” (p. 1).	(clinic* or practi* or primary or physician* or refer* or visit* or outpatient* or consult* or family or communit* or ambulatory or centre? or center? or office).ti,ab.

### Stage 2: Identifying relevant literature

Relevant studies for this review will be identified through searching electronic databases of the published literature which will include: Ovid MEDLINE, Ovid EMBASE, EBSCO CINAHL, Ovid PsycINFO, and the Wiley Cochrane Library. These five are frequently used databases for health sciences literature, therefore, suggested by the research librarian. However, this may be a limitation for this review as we do not plan to include any technology-specific database.

The search strategy for Ovid MEDLINE was first developed with support from the team’s information scientist (HC) and has been peer-reviewed using the Peer Review of Electronic Search Strategies (PRESS) tool. The MEDLINE search strategy is included in Additional file 1. This search strategy will be then translated over to the remaining databases. A “primary care” filter, which was developed and validated by Gill et al. [31] will be applied to the searches. The literature search will be limited to articles published after 2000; articles published before this date may be less relevant to the current ICT integrated primary care model landscape [32].

### Stage 3: Study selection

The study selection process will comprise two levels of screening: (1) a title and abstract review and (2) a full-text review. Both levels will be conducted in duplicate using the knowledge synthesis software Covidence. At both levels of screening, any discrepancies between reviewers will be discussed and resolved by the research team collaboratively. The PRISMA framework [33] will be used to illustrate the study selection process and the Preferred Reporting Items for Systematic Review and Meta-Analysis Protocols (PRISMA-P) extension for scoping review guidelines [34] (Additional file 2, Table 1) will be used to guide reporting of the study findings.

To be included the studies will meet the inclusion and exclusion criteria listed in Table 4. To ensure an included article is peer-reviewed, we will check whether the article is published in any peer-reviewed journals. As this scoping review targets general complex patients, we excluded cancer patients and patients with a mental disorder. Patients with cancer often require a different treatment pathway than non-cancer patients [35]. Similarly, patients with a mental disorder often receive a different treatment protocol and diagnostic procedure than their counterparts with biophysical conditions [36, 37]. Studies focused exclusively on individuals younger than 18 are also excluded as ICTs designed for these individuals involve parental consent and subsequently a unique approach to ICT adoption.

**Table 4:** Inclusion/exclusion criteria

Inclusion criteria	Exclusion criteria
1. Any published articles including quantitative, qualitative, mixed, or multi-methods research, including both comparative (e.g., randomized, controlled, cohort, quasi-experimental) and non-comparative (e.g., survey, narrative audit) methods, hand search of any relevant articles of systematic and scoping reviews	1. The target population for the study is individuals younger than 18.
2. The intervention has an ICT-enabled healthcare model or has an ICT component	2. The target population for the study is individuals with cancer or mental disorder.
3. The intervention is based on an integrated healthcare model or team-based care	
4.The intervention includes patients with complex care needs	
5. The intervention takes place within primary care or include primary care in the integrated care model.	

### Stage 4: Data extraction

All included studies will be reviewed and charted independently by six team members using a pilot-tested data abstraction form. Charting is a technique for organizing and interpreting data by sifting, categorizing, and sorting material, according to key issues and themes [29]. We will run the data abstraction pilot-test on a random selection of included articles to ensure consistency across reviewers. Necessary changes will be made and shared with the team before abstracting the remaining articles. The collected data will be stored and compiled in Microsoft Excel for data validation and coding. We will not appraise the methodological quality or risk of bias in the included studies which is consistent with scoping review guidelines [38]. Table 5 shows the data that will be extracted from the selected literature:

**Table 5:** Data extraction framework

Domain	Information to be extracted
Citation summary	• Author(s) • Title • Study citation • Research design • Study location
Characteristics of the patient population	• Age • Gender and/or sex • Type and number of health conditions • Any additional characteristics provided (i.e., marital status, education level, ethnicity)
Characteristics of the integrated primary care	• Setting (inpatient, outpatient, community-based or rehabilitative care) • Type of service providers (i.e., general physician, nurses)
Characteristics of the implemented technology	• Attributes of technologies (functionality, use, and roles of technologies in the given care setting) • Aims/purpose of the implemented technology
Facilitators and barriers to implementation	• Description of the factors that inhibit or facilitate the implementation of ICTs for complex patients in the integrated primary care model

### Stage 5: Data summary and synthesis of findings

The charted data will be organized, coded, and thematically analyzed. Based on the extracted data, each study will be coded based on (1) their primary function (i.e., delivery care tool, self-management support) and (2) the characteristics of integrated care settings (i.e., professional-level integration, clinical level integration). Using a deductive coding approach [39], the key elements of eCCM [17] will be used to determine the primary function of the identified ICTs, whereas RMIC [23] will be used to identify characteristics of the care setting (see Table 1). We will also report the results with descriptive statistics with absolute and relative frequencies. This descriptive synthesis approach aligns with scoping review guideline [38].

### Stage 6: Knowledge translation and stakeholder consultation

Levac et al. propose that the knowledge translation and consultation phase employ an opportunity for stakeholder engagement [30]. To address stakeholder involvement, we will share our preliminary research findings with external healthcare policymakers and researchers to validate search results and support knowledge translation efforts. Special interest group members of the International Foundation for Integrated Care (IFIC) will be consulted for expert opinions on our scoping review findings [40]. This special interest group consists of a wide range of membership with representation from research and academia, health care management, front line providers, government authorities/policymakers, patients, and families, as well as private stakeholders. Members of this special interest group have a background in the development, adoption, and/or evaluation of digital health solutions in models of integrated care. Furthermore, we will prepare educational materials and presentations to disseminate study findings to multi-disciplinary health teams, caregivers, and patients and at relevant national and international conferences. Results will also be published in a peer-reviewed journal and infographics will be developed for easy uptake by a wider user audience.

#### Anticipated timeline

The study is currently in the study selection process. Electronic database searches were completed till December 2021, which yielded 52, 128 articles. After title and abstract screening 293 studies were selected for full text review. Ultimately, 31 studies met the eligibility criteria. As the next step, we will conduct data extraction and data synthesis for the 31 studies. The authors anticipate that the results of the study will be submitted by September 2021.

## Discussion

To our knowledge, this is the first review that will comprehensively identify the existing ICTs used to support the health and social care needs of complex patients within an integrated primary care model. This scoping review will inform future research and key stakeholders about the important roles that these technologies play to improve patients’ and providers’ care experience. In addition, this review will identify potential gaps in the current ICT landscape that could improve care coordination, communication, and disease management among complex patients.

### Limitation

There are some limitations to this protocol. Consistent with the scoping review methodology, the scope of the research objective is broad [41]. However, we have chosen the scoping review method over systematic review because this study aims to identify relevant evidence and concepts on a broad and complex topic; meaning the definition and types of ICTs [21, 22] and the definition of integrated primary care [23] is heterogeneous; hence, it would be difficult to operationalize a systematic review on the topic. Additionally, we intend to include a heterogeneous study design to provide a comprehensive overview of relevant ICTs in the integrated primary care context. This approach is appropriate given the literature in ICTs and integrated care is still quite formative so we anticipate many studies at more developmental stages of research (such as exploratory and co-design studies) as opposed to clinical trials.

## Supplementary Information

**Additional file 1.** Search strategy-OVID MEDLINE.

**Additional file 2: Table 1**. Preferred Reporting Items for Systematic reviews and Meta-Analyses extension for Scoping Reviews (PRISMA-ScR) Checklist.

## Data Availability

Search strategies are available in Additional file 1.

## Abbreviations

ICTs: Information and Communication Technologies
RMIC: Rainbow Model of Integrated Care
eCCM: eHealth enhanced chronic care model
IFIC: International Foundation for Integrated Care
