# Supplementary material for: Information and Communication Technologies (ICTs) enabling integrated primary care for complex patients: a protocol for a scoping review
Source: Syst Rev. 2022 Sep 7;11:193. doi: 10.1186/s13643-022-02057-5 (PMC9450266; doi:10.1186/s13643-022-02057-5)
Supplement: Supplementary file 1 — Additional file 1. Search strategy-OVID MEDLINE. [file 13643_2022_2057_MOESM1_ESM.docx]

Database: Ovid MEDLINE: Epub Ahead of Print, In-Process & Other Non-Indexed Citations, Ovid MEDLINE® Daily and Ovid MEDLINE® <1946-Present>

1 exp "Delivery of Health Care, Integrated"/

2 "Continuity of Patient Care"/

3 exp Patient Care Planning/

4 exp Patient-Centered Care/

5 exp Patient Care Management/

6 Patient Care/

7 exp Interprofessional Relations/

8 ((Integrat* or multidisciplin* or interdisciplin* or interprofession* or team* or coordinat* or comprehensive or shar* or manage* or organi?ed or coop* or seamless or continu*) adj3 (care or healthcare or service* or deliver* or communicat* or relation* or treatment* or strateg* or program* or system*)).tw,kf.

9 Team*.tw,kf.

10 ((Case or cases or care or transition* or patient* or disease* or treatment*) adj3 (manage* or plan*)).tw,kf.

11 (patient adj3 (cent?ed or tailored or integrat* or orient* or focus*) adj3 care).tw,kf.

12 ((Linked or network* or structur*) adj3 care).tw,kf.

13 (Care adj3 (coordinat* or continu* or guid* or transmural)).tw,kf.

14 ((Critical or clinical) adj2 pathway*).tw,kf.

15 or/1-14 [integrated health care concept]

16 exp Technology/

17 exp Medical Records Systems, Computerized/

18 exp informatics/

19 exp computing methodologies/

20 exp Technology, Radiologic/

21 exp Telecommunications/

22 exp Management Information Systems/

23 exp Diagnosis, Computer-Assisted/

24 ((communicat* or health* or informat* or comput* or medical) adj3 (technol* or system* or applicat* or process*)).tw,kf.

25 (electronic adj3 record*).tw,kf.

26 (ehealth or electronic health or telehealth or tele-health or telemedicine or tele-medicine or telecommunicat* or tele-communicat* or videoconferenc* or video-conferenc* or virtual care or teleradio* or tele-radio* or telemetry or mobile app* or informatics or computer-assist* or computer assist* or mobile health or mhealth or m-health or software or EHR? or EMR?).tw,kf.

27 ((virtual or remote or distance or mobile or video) adj3 (consult* or health or medicine)).tw,kf.

28 or/16-26 [eHealth Technology concept]

29 exp Chronic Disease/

30 exp Comorbidity/

31 ((chronic* or complex or multi* or concurren* or co-occur* or co occur* or co-exist* or co exist* or dual or permanent or nonrevers* or non-revers*) adj2 (diagnos* or disease* or ill* or condition* or insufficienc* or disorder* or sick*)).tw,kf.

32 (multimorbid* or multi-morbid* or comorbid* or co-morbid* or CCC).tw,kf.

33 (poly-patholog* or polypatholog*).tw,kf.

34 (pluri-patholog* or pluripatholog*).tw,kf.

35 or/29-34 [Chronic illness concept]

36 15 and 28 and 35 [Integrated care concept + eHealth Technology concept + Chronic Illness concept]

37 exp Primary Health Care/

38 (clinic* or practi* or primary or physician* or refer* or visit* or outpatient* or consult* or family or communit* or ambulatory or centre? or center? or office).ti,ab. [Primary Care Search filter from Gill, at el Family Practice 31(6):739-745, 2014 ]

39 37 or 38 [Primary Care concept]

40 36 and 39 [three concepts with primary Care filter]

41 40 not ((exp infant/ or exp child/ or adolescent/) not exp adult/) [To remove studies indexed as child only]

42 limit 41 to yr="2000 -Current"
